# Anorectal manometry for the diagnosis of pelvic floor disorders in patients with hypermobility spectrum disorders and hypermobile Ehlers-Danlos syndrome

**DOI:** 10.1186/s12876-022-02572-8

**Published:** 2022-12-23

**Authors:** Wendy Zhou, Thomas A. Zikos, Houssam Halawi, Vipul R. Sheth, Brooke Gurland, Linda A. Nguyen, Leila Neshatian

**Affiliations:** 1 Department of Medicine, Division of Gastroenterology and Hepatology, Stanford University, 430 Broadway St. Pav C 3rd Floor, GI Suite, Redwood City, CA 94063 USA; 2 Department of Radiology, Stanford University, Redwood City, USA; 3 Department of Surgery, Stanford Universtiy, Redwood City, USA

**Keywords:** Pelvic floor disorders, Anorectal manometry, EhlersDanlos syndrome (EDS), Psychosomatic disorders

## Abstract

**Introduction:**

Functional gastrointestinal disorders (FGID) including impaired rectal evacuation are common in patients with Hypermobility Spectrum Disorder (HSD) or Hypermobile Ehlers-Danlos Syndrome (hEDS). The effect of connective tissue pathologies on pelvic floor function in HSD/hEDS remains unclear. We aimed to compare clinical characteristics and anorectal pressure profile in patients with HSD/hEDS to those of age and sex matched controls.

**Methods:**

We conducted a retrospective review of all FGID patients who underwent high resolution anorectal manometry (HR-ARM) and balloon expulsion test (BET) for evaluation of impaired rectal evacuation. Patients with HSD/hEDS were age and sex matched to a randomly selected cohort of control patients without HSD/hEDS. An abnormal BET was defined as the inability to expel a rectal balloon within 2 minutes. Wilcoxon rank sum test and Fisher’s exact test were used to make comparisons and logistic regression model for predictive factors for abnormal evacuation.

**Results:**

A total of 144 patients (72 with HSD/hEDS and 72 controls) were analyzed. HSD/hEDS patients were more likely to be Caucasian (*p* < 0.001) and nulliparous. Concurrent psychiatric disorders; depression, and anxiety (*p* < 0.05), and somatic syndromes; fibromyalgia, migraine and sleep disorders (*p* < 0.001) were more common in these patients. Rate of abnormal BET were comparable among the groups. HDS/hEDS patients had significantly less anal relaxation and higher residual anal pressures during simulated defecation, resulting in significantly more negative rectoanal pressure gradient. The remaining anorectal pressure profile and sensory levels were comparable between the groups. While diminished rectoanal pressure gradient was the determinant of abnormal balloon evacuation in non HSD/hEDS patients, increased anal resting tone and maximum volume tolerated were independent factors associated with an abnormal BET in HSD/hEDS patients. Review of defecography data from a subset of patients showed no significant differences in structural pathologies between HSD/hEDS and non HSD/hEDS patients.

**Conclusions:**

These results suggest anorectal pressure profile is not compromised by connective tissue pathologies in HSD patients. Whether concurrent psychosomatic disorders or musculoskeletal involvement impact the pelvic floor function in these patients needs further investigation.

## Introduction

Hypermobility Spectrum Disorder (HSD) and hypermobile Ehlers-Danlos Syndrome (hEDS) comprise a group of connective tissue disorders with symptomatology consisting of hypermobile joints, hyperextensible skin, frequent dislocations without a confirmed cause, resulting in chronic pain and psychological dysfunction. Symptoms of functional GI disorders (FGID) are commonly reported by patients with HSD/hEDS [1, 2].

Pelvic floor symptoms are reported by HSD/hEDS patients at a high prevalence [3]. Moreover, prevalence and severity of pelvic floor symptoms are increased in HSD/hEDS compared to patients with other connective tissue diseases and joint hypermobility syndromes [2, 3].

The most common pelvic floor symptoms in HSD/hEDS include incomplete bowel evacuation, symptoms suggestive of a functional defecation disorder, hemorrhoid, and proctalgia [4]. Evaluation of pelvic floor disorders using testing such as high resolution anorectal manometry (HR-ARM) and defecography have confirmed the presence of functional pathologies such as dyssynergic defecation and structural pathologies such as rectocele, and excessive perinium descent in HSD/hEDS patients [3]. In a study of gastrointestinal manifestations in hEDS, 10 patients were found to have elevated resting sphincter pressure and 18 were diagnosed with rectal evacuation disorder among 30 patients who had undergone HR-ARM for constipation [4]. Morphological abnormalities on dynamic barium defecography are shown to be significantly more common in patients with joint hypermobility compared to controls [5]. This suggest that structural abnormalities may be the pathophysiology for impaired rectal evacuation in patients with connective tissue disorders [5].

Despite the assumption of connective tissue abnormalities leading to pelvic floor laxity in HSD/hEDS, the exact pathoetiology of pelvic floor disorders in HSD/hEDS remains unclear [6]. Our study takes a closer look at pelvic floor abnormalities in patient with HSD/hEDS. The aim of the study was to compare clinical characteristics and anorectal pressure profile in patients with HSD/hEDS to those of age and sex matched patients without HSD/hEDS.

## Materials & Methods

### Patient Population

Electronic medical records of patients who completed HR-ARM between 2014 to 2019 at a tertiary care Gastroenterology clinic for evaluation of chronic constipation or impaired rectal evacuation, were screened for HSD/hEDS. The 2017 International EDS diagnostic classification was used for the diagnosis of hEDS [7]. HSD was diagnosed based on documented positive Beighton score and history of musculoskeletal involvement as defined by the Ehlers Danlos Society [8]. Patients with HSD/hEDS were age and sex matched 1:1 to non HSD/hEDS patients at random who completed HR-ARM for similar indications. Demographic data was collected, along with HR-ARM results, and relevant co-morbidities and symptoms. The study was approved by the institutional review board (#46918). Prior to testing, patients were advised to discontinue any cholinergic agents, promotility agents, stool softeners, or laxatives for 48–72 hours.

### High Resolution Anorectal Manometry (HR-ARM)

Patients were instructed to administer one fleet enema 2 hours prior to the HR-ARM procedure. Patient were asked to stop opiates prior to the tests. The procedure was performed in the left lateral position with patients’ knees and hips flexed. A 2-dimensional high-resolution solid-state anorectal manometry catheter (*Medtronic*, Minneapolis, MN, USA) was inserted into the rectum. Rectal propulsive pressures were captured from the proximal sensor located in the rectal balloon, and the average of pressures from anal sensors through the e-sleeve provided the anal pressures. With the catheter in place, patients were instructed to relax, squeeze, bear down, and cough according to previously reported protocol [9]. Resting pressures were recorded after 3 to 5 minutes of acclimation to the catheter. Squeeze pressures were obtained three times with 1 minute of rest in between. Each time, the patient was asked to hold the squeeze for 20 seconds. Patients were then instructed to push and bear down to simulate evacuation for 20 seconds (with a deflated rectal balloon). This was repeated twice with a 1-minute resting period in between. The rectoanal inhibitory and cough reflexes were assessed. Residual anal pressure was defined as the difference between the baseline pressure and the lowest (residual) pressure within the anal canal during attempted defecation. A low residual anal pressure indicates adequate anal relaxation during defecation. Rectoanal gradient was defined as the difference between rectal pressure and residual anal pressure; a positive rectoanal gradient indicates normal defecation.

Rectal sensation testing was performed by distending a rectal balloon in 10 ml increments until the first sensory threshold was reached, and in 30 ml increments thereafter until a maximum volume of 350 ml was reached, or until the patient reported severe urgency as defined by volume of urge to defecate (whichever was reached first). Analysis was done using the *ManoScan*^tm^ *V.3* (*Medtronic*, Minneapolis, MN, USA).

### Balloon Expulsion Test (BET)

Balloon expulsion was performed using a non-latex rectal balloon (*Mui Scientific*, Mississauga, ON, Canada). Balloon was inserted into the rectum while patient was in the left lateral decubitus position and then filled with 50 ml of water. Patients were asked to expel the balloon while sitting on a bedside commode. If patients could not expel the balloon in 2 minutes, it was deflated and removed manually. We chose 2 minutes as the upper limit of normal [10]. Although a cut off of 1 minute has been proposed for normal BET, maximum allowed expulsion time (between 1 and 5 minutes) did not appear to significantly affect test performance relative to reference tests based on a recent systemic review and meta-analysis [11].

### MR Defecography

Pelvic MR defecography was performed on 3-Tesla MRI systems (various vendors: GE, Siemens, and Philips) with use of a phased array torso coil with the patient in supine position, with the knee slightly flexed [12–14]. Patients self administered 1 enema 4 hours before arriving for imaging appointment. Before imaging, 200 mL of gel was placed in the rectum for visualization and to assess function during defecation phase imaging. Imaging evaluation included sagittal balanced steady-state free precession (SSFP) for dynamic assessment, resting T2-weighted fast spin echo sequences acquired in the axial and sagittal planes, axial T2-weighted 3D volumetric fast spin echo, coronal T2-weighted single shot fast spin echo at rest and with Valsalva in the coronal plane.

Standard pelvic MR defecography measurements at rest and maximal stress were collected including H and M line, rectocele size, Oxford grade of rectal prolapse, and grades of uterovaginal and bladder descent. Pubococcygeal line was drawn from the inferior border of the pubic symphysis to the last coccygeal joint and used as the reference line to measure organ prolapse. The H line was drawn from the inferior margin of the pubic symphysis to the posterior aspect of the anorectal junction, and represents the diameter of the levator hiatus. The M line was drawn perpendicularly from the posterior end of the H line to the pubococcygeal line, and represents the descent of the hiatus [12–14].

The grading of rectal prolapse was noted through Oxford classification. Grade 0 was defined as no rectal prolapse present. Grades 1–2 intrarectal intussusception to proximal border of the rectocele and into the rectocele respectively. Grades 3–4 intussusception were onto the top of the anal canal or into the canal respectively. Grade 5 was external prolapse through the anal verge. The staging of uterovaginal or bladder prolapse was defined based on the level of the prolapse of the bladder neck or posterior lip of the cervix with respect to the pubococcygeal line. Stage 0 was defined as no uterovaginal or bladder prolapse present. Stage 1, 2, and 3 were defined as bladder and/or uterine descent to 1–3 cm, 3–6 cm, or > 6 cm below the pubococcygeal line [12–14].

### Data Analysis

Our study compared clinical characteristics and anorectal manometry profile between patients with and without HSD/hEDS evaluated for constipation or impaired rectal evacuation. Characteristics collected included sex, race, co-morbidities, and GI symptoms. Anorectal manometry variables evaluated included sphincter pressures, simulated defecation, sensory levels. BET data was also compared.

Median pressure values and sensory level variables such as first sensation, defecation urge, and maximum tolerated volume and MR defecography measures were compared using Wilcoxon rank sum test. The percent with failed BET were compared using Fisher’s exact test.

Multiple logistic regression using stepwise forward elimination with a cutoff for model inclusion of *p* < 0.15 was used to evaluate for confounding. Included variables are listed in the results accordingly. All statistical analysis was done using Stata version 14 (*StataCorp LLC*, College Station, TX, USA). In all cases, *p* ≤ 0.05 was considered significant.

## Results

### Patient Demographics

We compared 72 individuals with HSD/hEDS to 72 age and sex matched individuals without HSD/hEDS, who underwent HR-ARM for evaluation of constipation or pelvis floor related symptoms. The majority of the individuals were female (96%). HSD/hEDS patients were more likely to be caucasian (*p* < 0.001). Table 1 compares demographics, co-morbidities and GI symptoms between the groups. While GI symptoms were common across both groups, they were significantly more common in HSD/hEDS.

**Table 1:** Baseline demographics

Variable	HSD/hEDS (***n*** = 72)	No HSD/hEDS (***n*** = 72)	***p***-value
**Basic Demographics**			
Age: Median (IQR)	36 (27–48)	36 (28–48)	1.000
BMI: Median (IQR)	22.9 (19.6–27.8)	24.6 (21.5–28.1)	0.080
Sex, n (% female)	69 (96)	69 (96)	1.000
Race, n (%)			**< 0.001**
Caucasian	62 (86)	36 (50)	
Black	0 (0)	2 (3)	
Hispanic	7 (10)	10 (14)	
Asian	2 (3)	12 (16)	
Native American	0 (0)	6 (8)	
**Obstetric History**			
History of Vaginal Delivery, n (%)	14 (20)	30 (43)	**0.003**
**Co-morbidities**			
Diabetes, n (%)	2 (3)	6 (8)	0.275
IBS, n (%)	41 (57)	36 (50)	0.504
Functional Dyspepsia, n (%)	22 (31)	11 (15)	**0.046**
Anxiety, n (%)	39 (54)	23 (32)	**0.011**
Depression, n (%)	37 (51)	20 (28)	**0.006**
Chronic Pain Syndrome, n (%)	27 (38)	4 (6)	**< 0.001**
Fibromyalgia, n (%)	38 (53)	13 (18)	**< 0.001**
Migraine, n (%)	38 (53)	13 (18)	**< 0.001**
Sleep Disorders, n (%)	45 (63)	18 (25)	**< 0.001**
Narcotic Use, n (%)	6 (8)	1 (1)	0.116
Chronic Fatigue Syndrome, n (%)	13 (18)	8 (11)	0.345
Mast cell activation syndrome, n (%)	7 (10)	2 (3)	0.166
Endometriosis, n (%)	10 (14)	6 (8)	0.427
**Gastrointestinal Symptoms**			
Abdominal Pain, n (%)	59 (82)	45 (62)	**0.015**
Nausea/Vomiting, n (%)	45 (63)	29 (40)	**0.012**
Dyspepsia, n (%)	51 (71)	26 (36)	**< 0.001**
Constipation, n (%)	70 (97)	61 (85)	**0.017**
Diarrhea, n (%)	28 (39)	25 (35)	0.730

Significant *p*<0.05 are in bold

### High Resolution Anorectal Manometry and Balloon Expulsion Test Findings

Table 2 summarizes the data collected from HR-ARM and BET. The anorectal pressure profile at rest and during squeeze, sensory levels, and rate of abnormal BET were comparable between the groups.

**Table 2:** Anorectal manometry test results

Variable	HSD/hEDS (***n*** = 72) Median (IQR)	No HSD/hEDS (***n*** = 72) Median (IQR)	***p***-value
Mean resting sphincter pressure (mm Hg)	80.0 (62.5–94.4)	74.0 (56.3–97.8)	0.73
Max resting sphincter pressure (mm Hg)	89.3 (72.2–105.0)	93 (65.8–119.1)	0.60
Max squeeze sphincter pressure (mm Hg)	166 (122.5–219.7)	146.5 (111.7–198.5)	0.13
Duration of squeeze (seconds)	19.1 (10.9–20.4)	16.7 (10.2–21.6)	0.96
**Simulated Defecation**			
Residual anal pressures (mm Hg)	73.8 (65.6–94.4)	63.7 (42.8–87.6)	**0.03**
% Relaxation during push	3 (−20–17.5)	8 (−3.5–27)	**0.04**
Intra-rectal pressure during push (mm Hg)	29.9 (13.4–51.1)	32.8 (20.0–55.4)	0.10
Rectoanal pressure gradient	−40.3 (−62.25- −19.5)	-19 (−52.9–32.9)	**< 0.001**
**Sensory levels**			
First sensation (mL)	40 (20–60)	40 (20–70)	0.10
Urge to defecate (mL)	75 (50–120)	70 (50–120)	0.25
Maximum tolerated (mL)	115 (80–180)	110 (75–160)	0.38
Failed Balloon Expulsion Test, n (%)	26 (36)	23 (32)	0.60
Dyssynergic defecation, n (%)	55 (76)	49 (68)	0.26
Rectal hyposensitivity, n (%)	28 (39)	26 (36)	0.73

*Abbreviations*: *IQR* Interquartile range

Significant *p*<0.05 are in bold

### Defecography Findings

**Fig. 1:**
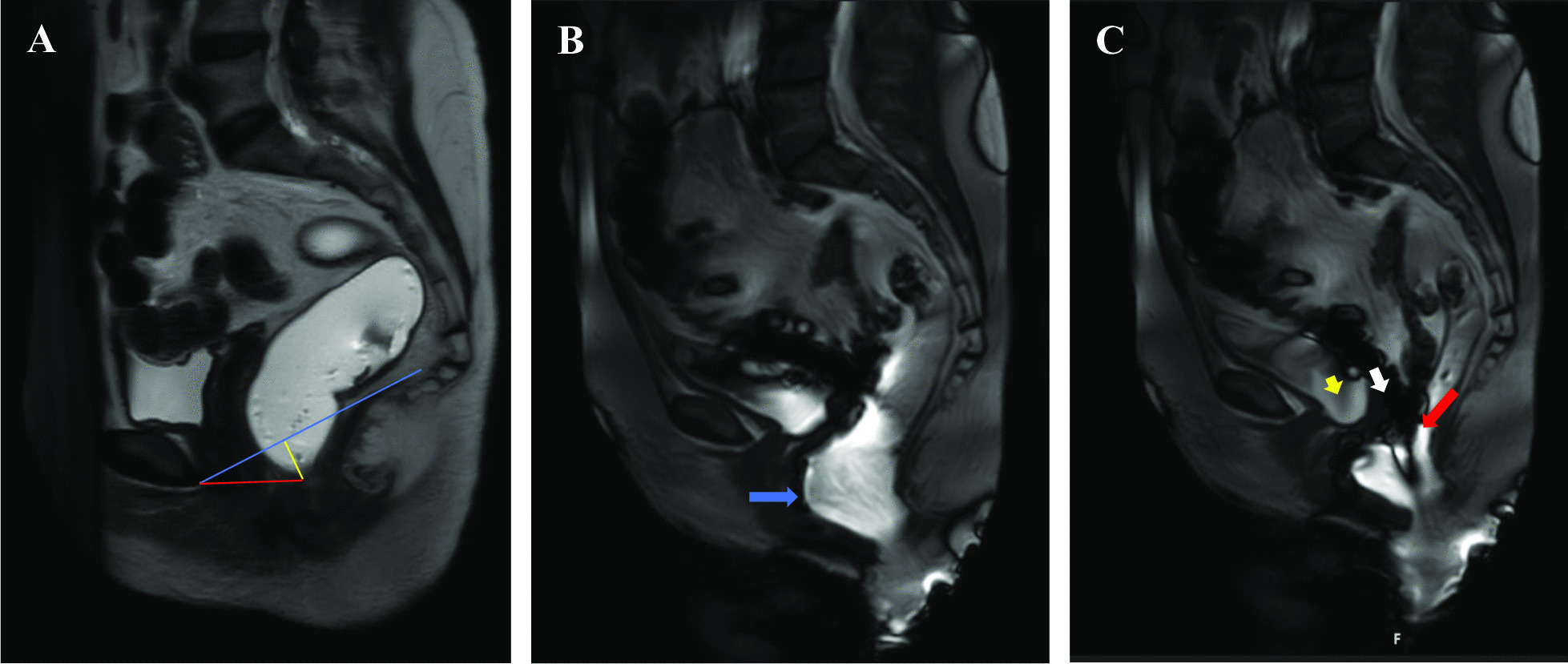
MR defecography illustrating examples of anatomical abnormalities during defecation. MR defecography illustrating examples of anatomical abnormalities during defecation (Example reference lines are shown: blue is PCL, red is H-line, yellow is M-line). **A** at rest, no pelvic floor relaxation or organ prolapse. **B** during initiation of defecation, notable for moderate anterior rectocele (blue arrow). **C** throughout defecation, notable for intra-anal (Oxford Grade 4) rectal intussusception, and Stage 1 bladder (red arrow) and vaginal (white arrow) prolapse (red arrow)

Table 3 summarizes MR defecography variables across both groups. 36 MR defecographies were performed and reviewed (16 HSD/hEDS and 20 non-HSD/hEDS). Multiple variables were highlighted including H and M lines (at rest and during strain), and structural abnormalities including rectoceles, uterovaginal, bladder, and rectal prolapses. There were no significant differences in resting and straining H and M lines across the groups. There was varying degrees of prolapses across both groups but without significant difference. Both groups had patients with contained rectoceles present. Figure 1 demonstrates structural abnormalities noted during MR defecography.

**Table 3:** Variables of MR defecography including structural lines, grading of rectal prolapses, and staging of uterovaginal and bladder prolapses

Variable		HSD/hEDS (***N*** = 16)	Non-HSD/hEDS (***N*** = 20)	***p***-value
**Rest; Median (IQR)**	H line	4.80 (4.25–5.95)	5.20 (4.45–6.25)	0.48
	M line	1.60 (1.10–2.90)	1.60 (0.70–2.50)	0.56
**Strain, Median (IQR)**	H line	6.40 (5.10–7.55)	6.55 (5.40–8.50)	0.58
	M line	3.80 (2.65–5.45)	3.15 (2.50–4.90)	0.50
**Oxford Grade of Rectal Prolapse, n (%)**	0	7 (44)	8 (40)	0.35
	1	0 (0)	2 (10)	
	2	3 (19)	4 (20)	
	3	3 (19)	5 (25)	
	4	3 (19)	0 (0)	
	5	0 (0)	1 (5)	
**Rectocele Size, n (%)**	Small	9 (56)	11 (55)	0.48
	Moderate	1 (6)	3 (15)	
	Large	2 (12)	0 (0)	
**Contained Rectocele, n (%)**		2 (12)	3 (15)	0.58
**Stage of Uterovaginal Descent, n (%)**	0	11 (69)	12 (60)	0.63
	1	5 (31)	6 (30)	
	2	0 (0)	2 (10)	
	3	0 (0)	0 (0)	
**Stage of Bladder Descent, n (%)**	0	13 (81)	16 (80)	1.00
	1	2 (12)	2 (10)	
	2	1 (6)	2 (10)	
	3	0 (0)	0 (0)	

*Abbreviations*: *IQR* Interquartile range

### Determinants of Abnormal Evacuation

Table 4 outlines multiple variables in anorectal manometry testing and evaluates potential determinants of abnormal balloon evacuation using multiple regression analysis. The following variables were included: mean resting pressure, maximum squeeze pressure, duration of squeeze, residual anal pressures, percent anal relaxation, and rectoanal pressure gradient during simulated defecation, and volume at first sensation, urgency, and discomfort. Only variables with *p* < 0.15 during standard linear regression were included in the multiple regression model. In all patients, higher rectoanal pressure gradient during simulated defecation, and lower volume at maximum tolerated volume were determinants of normal rectal balloon evacuation. When separated, the groups in non HSD/hEDS patients, higher rectoanal pressure gradient during simulated defecation and in patients with HSD/hEDS lower resting anal pressures (anal tone) and volumes at maximum tolerated levels, were independent factors associated with normal rectal balloon evacuation.

**Table 4:** Determinants of abnormal rectal balloon evacuation using multivariable logistic regression with forward elimination

Variable	Adjusted OR (95% CI)	***p***-value
**All patients**		
Rectoanal pressure gradient on simulated defecation	1.010 (1.001–1.020)	**0.03**
Maximum tolerated volume	0.993 (0.988–0.998)	**0.007**
Duration of sustained squeeze	0.954 (0.899–1.012)	0.12
**Non HSD/hEDS patients**		
Rectoanal pressure gradient on simulated defecation	1.032 (1.009–1.057)	**0.009**
Residual anal pressure	1.020 (0.993–1.047)	0.16
**HSD/hEDS patients**		
Rectoanal pressure gradient on simulated defecation	0.996 (0.981–1.012)	0.64
Maximum tolerated volume	0.988 (0.981–0.996)	**0.004**
Mean resting sphincter pressure (Anal tone)	0.968 (0.939–0.998)	**0.034**
Max squeeze sphincter pressure (Anal contractility)	1.008 (0.998–1.018)	0.14

*Abbreviations*: *OR* Odds-ratio, *CI* Confidence interval

Significant *p*<0.05 are in bold

## Discussion

Both functional and structural pelvic floor pathologies have been reported in patients with HSD/hEDS [1, 15]. This is the largest study to use HR-ARM with BET in the evaluation of pelvic floor disorders in patients with HSD/hEDS. We hereby showed with HR-ARM data that pelvic floor function in HSD/hEDS, as characterized by anal resting and squeeze pressures, was comparable to that of age and sex matched controls undergoing HR-ARM for similar indications. However, HSD/hEDS patients showed significantly less anal sphincter relaxation associated with higher residual anal pressures and therefore more negative rectoanal pressure gradient on simulated defecation. These pressure patterns on HR-ARM may provide mechanistic information to better understand specific pathophysiologic pathways leading to pelvic floor disorders in HSD/hEDS. Future studies are needed to better define these specific manometric patterns on HR-ARM in patients with HSD/hEDS. If identified and validated, these manometric patterns could serve as potential biomarkers to differentiate pelvic floor disorders in HSD/hEDS from those in non HSD/hEDS, thus acting as targets for individualized biofeedback therapies tailored to these specific manometric abnormalities.

In contrast to the common assumption of pelvic floor laxity in these patients, anal sphincter pressures at rest and/or upon squeeze were comparable across both groups. This was also noted in the Mayo Clinic cohort of hEDS patients where 10 out of 18 patients diagnosed with rectal evacuation disorder (of a total of 30 patients who underwent HR-ARM) had elevated resting tone, and three had abnormally high squeeze pressures [4]. Overall frequency of sphincter abnormalities were similar among patients with or without HSD [9]. Our study results, along with prior reports, suggest multifactorial pathophysiology of pelvic floor disorders in HSD/hEDS.

Functional defecatory disorders often involve impaired relaxation or inappropriate contraction of pelvic floor muscles with or without inadequate propulsive forces during evacuation [16, 17]. Median rectoanal pressure gradient was significantly more negative in patients with HSD/hEDS (− 40.3 vs − 19, *p* < 0.001) mainly from inadequate anal relaxation (*p* = 0.038), and therefore higher residual anal pressures (*p* = 0.030), rather than from differences in rectal propulsive forces. These results were consistent with findings from previous studies [17]. Using 2 abnormal sensory levels, based on the published normal values [10] for the diagnosis of rectal hyposensitivity we showed that nearly 40% of hEDS/HSD patients had rectal hyposensitivity which is comparable to the data from Choudhary et al. [18]. The rate of rectal hyposensitivity in the non hEDS/HSD group was also high at 36% which is likely to be related to the characteristics of patients population in our cohort [19]. Large observational studies have shown that reduced rectal sensation is much more common in patients with constipation and corelates with worsening of constipation severity [19, 20]. Rectal hyposensitivity is shown to be associated with abnormal balloon expulsion test in patients with constipation suggesting a functional pathology in those patients with evacuation disorder [19]. Our findings further underscores the importance of rectal sensory function for normal rectal evacuation in patients with hEDS/HSD.

The rate of abnormal BET was not significantly different between the groups, with around 30% of patients having an abnormal BET. However, our data suggested different potential predictors of abnormal BET (as surrogate marker for abnormal rectal evacuation) in HSD/hEDS compared to non HSD/hEDS patients. In HSD/hEDS patients, higher resting sphincter pressures and rectal sensory level for maximum tolerated volume were associated with abnormal BET, while a more negative rectoanal gradient was associated with abnormal BET in non HSD patients. It is important however to recognize that, while statistically significant, these determinants had an odds ratio approaching 1, suggesting minimal clinical effect on the actual defecatory function. This further highlights the multifactorial nature of the pathophysiology of pelvic floor dyssynergy in general, and in patients with HSD/hEDS in particular. We speculate that, while these determinants may only play a minor role in specific pathophysiologic mechanisms, they may serve as diagnostic clues to differentiate pelvic floor disorders in patients with HSD/hEDS. Further prospective research is needed to better understand the various pathophysiologic mechanisms associated with an abnormal BET and leading to pelvic floor disorders in patients with HSD/hEDS.

In our study, only a small number of patients had defecography, and we noted no significant difference in pelvic floor relaxation between the two groups. Furthermore, there was no significant difference in the rate of structural pathologies among the groups, which is consistent with findings from a recent study by Choudhary et al. [18]. This data remains insufficient to determine the exact prevalence of these anatomical abnormalities in HSD/hEDS compared to non HSD/hEDS, and further research is needed.

In all cases, current general consensus is that these anatomical abnormalities are caused by underlying functional pathologies for which conservative non-surgical management is considered first line therapy [21]. Studies have shown significant improvement in the stage and severity of these anatomical abnormalities, and in symptoms with use of pelvic floor therapy alone [21]. Avoiding unnecessary surgeries is especially important in patients with HSD/hEDS, as the high burden of GI symptoms and psychosomatic disorders are shown to negatively impact their quality of life and increase health care utilization [22, 23].

Consistent with previous studies, the majority of our patients with HSD/hEDS were female and Caucasian [24]. Somatic disorders are known to be associated with FGID in patients with HSD/hEDS. In our cohort, the burden of GI symptoms and prevalence of psychosomatic disorders were significantly higher in HSD/hEDS patients, and increased the likelihood of HSD/hEDS diagnosis. Moreover, the rates of anxiety and depression were overall high among the patients of this cohort. These psychiatric and somatic disorders were noted at a significantly higher rate in HSD/hEDS patients when compared to non HSD/hEDS counterparts. While the differences among the groups are unique to this cohort, numbers are consistent with previous reports of psychiatric disorders in more than half of patients with hEDS [25]. Previous studies have suggested that somatization is a confounder of FGID in HSD/hEDS patients [26]. Neverthless, the odds of FGIDs in this population is significantly reduced after adjusting for somatic symptoms [1].

Mental stressors are shown to increase anal pressures in constipated women [27] and it is conceivable that they could act as confounders in pelvic floor disorders. Future studies are needed to assess the impact of psychosomatic disorders on the pathophysiology of anorectal disorders in HSD/hEDS patients.

We Found a Higher prevalence of somatic disorders in patients with HSD/hEDS with pelvic floor complaints. Psychosomatic disorder are associated with a greater perception of pain and a heightened sensitivity, a phenomenon called central sensitization [28]. Patients with psychosomatic disorders commonly report multiple symptoms, including bloating, or abdominal pain, which also overlap with symptoms of pelvic floor disorders in HSD/hEDS. It is thus conceivable that psychosomatic factors could play a role in the symptomatology of pelvic floor disorders in patients with HSD/hEDS, through central sensitization and abnormal symptom perception. Further investigation is needed to evaluate the potential role of central sensitization and psychosomatic disorders in the etiology and management of symptoms of pelvic floor disorders in HSD/hEDS.

Our study is limited by its retrospective nature. Patient symptoms were not obtained using validated questionnaires, but from chart review, thus introducing potential error. Furthermore, the study was conducted at a tertiary referral center, which increases the risk of selection and referral bias. To minimize errors, we age- and sex- matched our cohorts, and used multiple logistic regression to identify potential confounders. Finally, our study is limited by a sample size of 144 patients, and future large prospective multicenter studies are needed to investigate pelvic floor disorders in HSD/hEDS. In particular, future prospective studies are required to focus on anorectal pathologies using physiologic testings and dynamic imaging, and to assess for cofounding psychiatric and somatic pathologies using validated questionnaires.

In conclusion, HR-ARM including rectal sensation testing and BET provided valuable information about pelvic floor motor and sensory function in patients with HSD/hEDS. Anal tone and contractility as well as percent of patients with dyssynergic defecation, rectal hyposenstivity and abnormal BET were comparable among the age and sex matched patients with or without HSD/hEDS. Future research of HR-ARM in patients with HSD/hEDS may help identify specific manometric patterns as potential biomarkers to guide the diagnosis and targeted therapy of pelvic floor disorders in HSD/hEDS.

## Data Availability

All data generated or analyzed during this study are included in this published article and its supplementary information files.

## Abbreviations

BET: Balloon expulsion test
BMI: Body mass index
FGID: Functional gastrointestinal disorders
GI: Gastrointestinal
hEDS: EhlersDanlos Syndrome
HR-ARM: High resolution anorectal manometry
HSD: Hypermobility spectrum disorder
IBS: Irritable bowel syndrome
MRI: Magnetic resonance imaging
OR: Odds ratio
SSFP: Steady-state free precession
